# Sleep Analysis in Adult *C. elegans* Reveals State-Dependent Alteration of Neural and Behavioral Responses

**DOI:** 10.1523/JNEUROSCI.1701-20.2020

**Published:** 2021-03-03

**Authors:** Daniel E. Lawler, Yee Lian Chew, Josh D. Hawk, Ahmad Aljobeh, William R. Schafer, Dirk R. Albrecht

**Affiliations:** ^1^Department of Biomedical Engineering, Worcester Polytechnic Institute, Worcester, Massachusetts 01609; ^2^Illawarra Health and Medical Research Institute and School of Chemistry and Molecular Bioscience, University of Wollongong, Wollongong 2522, Australia; ^3^Department of Neuroscience, Yale University School of Medicine, New Haven, Connecticut 06536-0812; ^4^Neurobiology Division, Medical Research Council (MRC) Laboratory of Molecular Biology, Cambridge CB2 OQH, United Kingdom; ^5^Department of Biology and Biotechnology, Worcester Polytechnic Institute, Worcester, Massachusetts 01609

**Keywords:** arousal threshold, calcium imaging, closed-loop stimulation, microfluidics, sensory processing, sleep dynamics

## Abstract

Sleep, a state of quiescence associated with growth and restorative processes, is conserved across species. Invertebrates including the nematode *Caenorhabditis elegans* exhibit sleep-like states during development, satiety, and stress. Here, we describe behavior and neural activity during sleep and awake states in adult *C. elegans* hermaphrodites using new microfluidic methods. We observed effects of fluid flow, oxygen, feeding, odors, and genetic perturbations on long-term sleep behavior over 12 h. We developed a closed-loop sleep detection system to automatically deliver chemical stimuli to assess sleep-dependent changes to evoked neural responses in individual animals. Sleep increased the arousal threshold to aversive stimulation, yet the associated sensory neuron and first-layer interneuron responses were unchanged. This localizes adult sleep-dependent neuromodulation within interneurons presynaptic to the premotor interneurons, rather than afferent sensory circuits. However, sleep prolonged responses in appetitive chemosensory neurons, suggesting that sleep modulates responsiveness specifically across sensory systems rather than broadly damping global circuit activity.

**SIGNIFICANCE STATEMENT** Much is known about molecular mechanisms that facilitate sleep control. However, it is unclear how these pathways modulate neural circuit-level sensory processing or how misregulation of neural activity contributes to sleep disorders. The nematode *Caenorhabditis elegans* provides the ability to study neural circuitry with single-neuron resolution, and recent studies examined sleep states between developmental stages and when stressed. Here, we examine an additional form of spontaneous sleep in adult *C. elegans* at the behavioral and neural activity levels. Using a closed-loop system, we show that delayed behavioral responses to aversive chemical stimulation during sleep arise from sleep-dependent sensorimotor modulation localized presynaptic to the premotor circuit, rather than early sensory circuits.

## Introduction

Sleep is a physiological state during which voluntary muscle activity ceases, sensory processing is modulated (Velluti, 1997), and anabolic, growth, and restorative processes occur in the brain and other tissues (Adam and Oswald, 1977). Sleep is observed across species, from mammals to invertebrates (Campbell and Tobler, 1984), where it controls energy usage and metabolism (Schmidt, 2014), macromolecular biosynthesis (Mackiewicz et al., 2007), and neural plasticity and memory consolidation (Frank and Benington, 2006). Owing to these critical functions, sleep deficiencies are associated with impaired cognitive function, productivity (Rajaratnam et al., 2013), and immune response (Luyster et al., 2012) and increased prevalence of cardiovascular disease (Newman et al., 2000), diabetes (Gottlieb et al., 2005), and obesity (Hasler et al., 2004). The initiation and cessation of sleep is mediated in most species by circadian rhythms which are controlled by environmental factors (Reppert and Weaver, 2002) and timing genes that are generally conserved across species (Panda et al., 2002). Several molecular pathways (Yamuy et al., 1999; Kramer et al., 2001; Siegel, 2004; Saper et al., 2005; Sehgal and Mignot, 2011; Tsunematsu et al., 2011; Weber et al., 2015) are involved in promoting sleep states and inhibiting arousal behavior, but it is currently unclear how these pathways modulate neural circuit-level sensory processing during sleep states (Hennevin et al., 2007), and how misregulation of neural activity may contribute to sleep disorders.

The nematode *Caenorhabditis elegans* provides distinct advantages for direct observation of neurologic function in freely-behaving animals. They are small (<1 mm), exhibit short generational times, and have a compact and fully mapped connectome of 302 neurons in hermaphrodites. Noninvasive optical measurements of neural activity can be made in living, behaving animals via genetically-encoded fluorescent calcium indicators such as GCaMP (Tian et al., 2009), and genetic tools are available for rapid generation of mutants and transgenic strains for mechanistic studies (Antoshechkin and Sternberg, 2007; Boulin and Hobert, 2012; Friedland et al., 2013). *C. elegans* demonstrate states of quiescence during lethargus between larval stages (Raizen et al., 2008; “developmentally-timed sleep”) and during periods of stress (Hill et al., 2014; “stress-induced sleep”), satiety (You et al., 2008; Gallagher and You, 2014), starvation (McCloskey et al., 2017; Skora et al., 2018), and hypoxia (Nichols et al., 2017). Additionally, adult *C. elegans* undergo quiescent periods after 1–2 h of swimming in liquid (Ghosh and Emmons, 2008) and in microfluidic chambers with open and constrictive geometries (Gonzales et al., 2019). Developmentally-timed and stress-induced quiescent states share fundamental sleep functions with other species (Singh et al., 2013), including processing of synaptic plasticity (Dabbish and Raizen, 2011) and metabolic control (Driver et al., 2013). They also share typical behavioral characteristics such as increased arousal threshold (Raizen et al., 2008; Cho and Sternberg, 2014), stereotypical posture (Schwarz et al., 2012; Iwanir et al., 2013; Tramm et al., 2014), homeostatic response to sleep deprivation (Raizen et al., 2008; Driver et al., 2013; Nagy et al., 2014a), and rapid reversibility (Raizen et al., 2008; Trojanowski et al., 2015).

*C. elegans* sleep has been observed using a variety of experimental platforms, including agar (Raizen et al., 2008) or agarose pads (Turek et al., 2015; Churgin et al., 2017) and microfluidic chambers that house individual animals (Singh et al., 2011; Nagy et al., 2014b; Huang et al., 2017; Gonzales et al., 2019) throughout multiple development stages. Neural activity measurements typically require immobilization by agarose pads (Spies and Bringmann, 2018) or microfluidic traps (Cho and Sternberg, 2014), which limit their use to developmentally-timed or induced sleep studies. However, adult sleep events occur spontaneously and are identified by analysis of locomotion and quiescent behaviors. Thus, to assess the functional circuit changes that occur during adult sleep, new methods for monitoring sleep state and stimulated neural responses in freely-moving animals are needed.

Here, we demonstrate two systems to quantify the behavioral and neural characteristics of sleep in young adult *C. elegans*. We first show that sleeping behavior exhibited by young adult *C. elegans* follows characteristic dynamics over 12 h in microfluidic devices and is altered by fluid flow, oxygen, bacterial food, food signals, and genetic perturbations affecting sensory input. Next, using a closed-loop chemical stimulation system, we observed an increased arousal threshold during adult sleep states, as has been observed previously in developmentally-timed sleep (Raizen et al., 2008), and simultaneously monitored neural activity via fluorescence microscopy during these behavioral responses. A sleep-dependent delay in response to aversive stimulation corresponded to diminished and delayed responses in premotor interneurons. However, responses in associated sensory neurons and first-layer interneurons were not modulated by sleep, localizing sleep-state neural circuit modulation within interneurons of the aversive sensorimotor subcircuit. These results suggest that sleep specifically alters the linkage between sensory stimuli and premotor neurons without changing upstream sensory or interneuron information. In contrast, responses in the appetitive sensory neurons were prolonged during sleep, indicating that sleep can affect sensory modalities differently. Together, these results illustrate that sleep modulates neural activity differently across stimuli and validate an experimental system to further dissect the molecular processes that produce this specificity.

## Materials and Methods

### 

#### Strains and *C. elegans* culture

All *C. elegans* strains were maintained under standard conditions on NGM plates and fed OP50 *Escherichia coli* bacteria seeded onto each plate. Wild-type animals were Bristol strain (N2). The following mutant strains were used: CB1611, *mec-4 (e1611)*; FK103, *tax-4 (ks28)*; CX32, *odr-10 (ky32)*; IB16, *ceh-17 (np1)*. Neural imaging strains expressing GCaMP in specific neurons were: (AWA; Larsch et al., 2013) CX14887, *kyIs598* [*gpa-6::GCaMP2.2b*]; (ASH; Larsch et al., 2013) CX10979, *kyEx2865* [*Psra-6::GCaMP3; Pofm-1p::GFP*]; (AIB) DCR6035, *olaIs94* [*Pinx-1::GCaMP6f; Punc-122::GFP*]; (AVA) QW607, *zfls42* [*Prig-3::GCaMP3::SL2::mCherry*] gifted by the Alkema lab; (RIS) AQ4064, *ljEx1119* [*Pflp-11::GCaMP3::SL2-tagRFP;unc-122::rfp*]. To make the RIS imaging line, a 2643 bp region immediately upstream of the ATG of the *flp-11* gene was amplified, similar to previously reported methods (Turek et al., 2016). This promoter was shown to express consistently in RIS and occasionally in other neurons (Turek et al., 2016). To synchronize for age, we picked L4 larval stage animals 1 d before experimentation such that all animals tested were at the young adult stage.

Animals were transferred to unseeded NGM plates immediately before experimentation. The plates were then flooded with the control buffer used for their respective experiment: S. basal buffer (100 mm NaCl, and 50 mm KPO_4_; pH 6.0) for unfed behavioral experiments and diacetyl stimulus experiments, S. medium buffer (1-l S. basal, 10 ml 1 m potassium citrate pH 6.0, 10 ml trace metals solution, 3 ml 1 m CaCl_2_, and 3 ml 1 m MgSO_4_) for feeding experiments, or a saline buffer (80 mm NaCl, 5 mm KCl, 20 mm D-glucose, 10 mm HEPES, 5 mm MgCl_2_, and 1 mm CaCl_2_; pH 7.2) for copper chloride stimulus experiments. Animals were then collected into loading tubing using a 1-ml syringe before injection into the microfluidic arena (Lagoy and Albrecht, 2015).

#### Microfluidic device fabrication

“Population behavior” and “neural imaging” microfluidic devices were fabricated as previously described (Lagoy and Albrecht, 2015). Briefly, transparency photomasks were printed at 25 000 dpi from designs sketched using DraftSight CAD software. SU-8 mold masters were prepared on silicon wafers using standard photolithography techniques, and microfluidic devices were fabricated by pouring degassed PDMS (Sylgard 184, Dow Corning) onto the mold and heat curing. Individual devices were then cut out and punched to provide inlet and outlet flow. A hydrophobic glass substrate was created by vapor deposition of tridecafluoro-1,1,2,2-tetrahydrooctyl trichlorosilane (TFOCS; Gelest) and then sealed reversibly to the microfluidic channels. An upper glass slide, with holes drilled over inlet and outlet ports with a diamond-coated drill bit, was sealed above the device, which was then was placed into a metal clamp.

#### Stimulus preparation

All odor dilutions were freshly prepared on the day of experimentation. NA22 *E. coli* stock solutions were prepared using previously described methods (Keil et al., 2017). Briefly, NA22 *E. coli* was cultured, concentrated into pellet form, and suspended in S. medium buffer. A stock solution was diluted to an OD600 of 7.0, and 50 µg/ml of kanamycin was added to prevent bacteria from growing. Chemical solutions were prepared at a 1:20 dilution of stock solution and filtered through a 5-µm filter. Diacetyl (1.1 μm) was prepared from a 10^−3^ dilution (11 mm) stock solution immediately before experimentation. Serotonin was prepared by dissolving serotonin creatine sulfate monohydrate powder (Sigma). Sodium sulfite (Sigma) solution was prepared moments before experimentation at 30 mm. We found that a 30 mm sodium sulfite solution would remain at nearly 0% oxygen with stirring for 12 h and without stirring for 5 d (Ocean Optics Neofox O_2_ probe kit), so the testing solution would be devoid of oxygen for entire 12-h testing period. The control solution of sodium sulfate was created by allowing for reoxygenation of the sodium sulfite solution for >5 d. For neural imaging experiments, 1 mm copper chloride solution was prepared the day of the experiment using copper chloride powder.

#### Microfluidic device setup

Microfluidic devices were cleaned, assembled, and degassed in a vacuum desiccator for 30–60 min before experimentation. Degassing devices accelerates the absorption of air bubbles within the device. For behavioral experiments, devices were filled with 5% (w/v) Pluronic F127 through the outlet port to prevent bacterial and molecular absorption by passivation of the microfluidic surfaces and to minimize bubble entrapment via its surfactant properties. Neural imaging devices were filled with control buffer alone. Reservoirs of loading solutions were prepared as previously described (Lagoy and Albrecht, 2015), purging the reservoir system of bubbles and connecting the tubing into the inlets of the device. Once flow was properly established, animals were gently loaded into their respective arenas and allowed to roam for 15–20 min before experimentation. For neural imaging experiments, a control valve was used to switch between stimulus and control buffer conditions within 0.5 s (Extended Data Fig. 7-1).

#### Population behavior imaging and identification of sleep events

Videos of population behavior were captured using a 6.6 MP PixelLink FireWire Camera at 1 fps for 12 h with an image resolution of ∼30 pixels/mm. Videos were processed after experimentation as previously described using MATLAB to extract behavioral data (Albrecht and Bargmann, 2011), and then further analyzed to identify sleep events. A minimum sleep entry window of 20 s and exit window of 5 s were used to quantify state transitions. To verify accuracy in parameters for sleep detection, user observed behavioral state was compared with script-calculated state on randomly chosen 60 s traces of an individual animals (Table 2). All behavior data were collected using “population behavior” devices with four 16 × 15 mm arenas capable of housing ∼25 animals per arena for simultaneous study.

#### Neural calcium imaging, sleep detection, and data analysis in closed-loop system

Closed-loop neural imaging videos were acquired at 5× magnification (NA = 0.25) with a Hamamatsu Orca-Flash 4.0 sCMOS camera using MicroManager/ImageJ software. The system has a green (λ = 520–550 nm) LED mounted overhead to provide pulsed brightfield illumination for tracking animal behavior and a Lumencor SOLA-LE solid-state lamp pulsed to excite GCaMP during fluorescence calcium imaging. To achieve autonomous experimentation for a closed-loop system, custom Arduino, MicroManager, and ImageJ scripts work together to control illumination timing, image acquisition, stimulus delivery, and sleep/wake state identification. An Arduino Uno microcontroller was programmed to control fluidic valves through a ValveLink 8.2 (AutoMate Scientific) controller and to control illumination sources for brightfield and fluorescent imaging. A MicroManager script allows the user to configure all camera and illumination settings before experimentation as well as all testing conditions for sleep assessment. Once the experiment is underway, the script initiates brightfield image capture at the desired framerate, and analyzes movement compared with the prior image in real time to determine the animal's behavioral state. If the current state and timing match the desired and preprogrammed conditions for neural imaging, the script initiates a fluorescence image stack recording and communicates with the Arduino via serial commands to control epifluorescence illumination and chemical stimulation with the desired timing.

Tracking of behavior of a single animal in the closed-loop neural imaging system was done using brightfield illumination with images captured at 0.1 fps. The current sleep/awake state of the animal was determined by an ImageJ script which calculates a movement index for each frame, represented as the fraction of body pixels moved since the previous frame, ranging from 0 to 1. A sleep state was defined as movement below the empirically-optimized threshold (0.125) for three consecutive frames (i.e., for 20–30 s). Optimization of detection parameters was done by maximizing accuracy from user observed behavioral states to script calculated states (Table 3). 1 min of consistent sleep or wake state frames were used to increase confidence in the animals' current state before neural imaging.

Calcium imaging was performed on freely-moving animals as previously described (Larsch et al., 2013) using lines expressing GCaMP in selected neurons. Neural activity was recorded in RIS neurons at 2 fps with no stimulation from the closed-loop system; however, motion was detected postprocessing to identify sleep bouts. Calcium imaging in ASH, AIB, AVA neurons was performed at 10 fps, using closed-loop stimulation to record responses to 10-s chemical stimulation from 5 to 15 s within a 30-s trial. Calcium imaging in AWA neuron was performed similarly, but was initiated every 5 min without closed-loop monitoring; sleep/wake state at stimulus onset was determined postcapture. Videos were analyzed for neural fluorescence and locomotion using NeuroTracker software in ImageJ, which tracks the position of the neuron over time and integrates fluorescent intensity of the soma using a 4 × 4 pixel box for ASH, AIB, and AVA neurons, and an 8 × 8 pixel box for the AWA neuron (Larsch et al., 2013). Fluorescence (F) was normalized by dividing by the initial baseline fluorescence in the first 4 s of each trial before stimulation (F_0_). As AIB fluorescence may not be at baseline at the beginning of each trial, baseline AIB intensity was determined for each animal across all trials, and individual AIB traces were excluded when animals engaged in reversal behavior immediately before stimulation. Traces and peak data from ASH, AIB, and AVA fluorescence are represented as 1-s moving average. Traces and peak data from AWA fluorescence is represented as a 0.3-s binned average through the 30-s trials.

The timing of arousal response was defined by the first frame of reversal movement for aversive stimuli, and by the first frame of head movement in sleeping animals stimulated with diacetyl. The onset of neural response was defined as the first frame three standard deviations above the prestimulation noise level.

#### Experimental design and statistical analyses

Sample sizes for each experiment are listed in the figure legends. All animals tested were adult hermaphrodites. Statistics were performed using one-way ANOVA with Bonferroni's correction for multiple comparisons or an unpaired two-tailed *t* test when specified for two sample comparison, using the Statistics and Machine Learning Toolbox in MATLAB. Data are represented as mean ± SEM unless otherwise stated. In behavioral experiments, animals were excluded when valid behavioral tracks comprised <8% of recording time, indicating an animal was not viable or not present during the test. In neural recordings, the top and bottom 1% of instantaneous fluorescent intensity was removed to reduce noise in peak fluorescence calculations. Complete statistical data for all figures reported in Table 1.

**Table 1.** Detailed statistical analysis

Figure	Test	*Post hoc* comparison
3*C*	Unpaired two-tailed *t* test	
	Hours 2–6: *t* = −2.825; df = 6; *p* = 0.03	
	Hours 6–12: *t* = 6.371; df = 10; *p* = 8.13E-05	
3*D*	Unpaired two-tailed *t* test	
	Hours 2–6: *t* = 2.797; df = 6; *p* = 0.031	
	Hours 6–12: *t* = −7.598; df = 10; *p* = 1.84E-05	
4*B*	One-way ANOVA	
	Sleep fraction: *F*_(1,185)_ = 336.3, *p* = 1.74E-43	
	Awake bout duration: *F*_(1,4228)_ = 65.4, *p* = 8.0E-16	
	Sleep bout duration: *F*_(1,5802)_ = 194.1, *p* = 2.02E-43	
4*C*	One-way ANOVA	
	Hour 1: *F*_(1,167)_ = 46.7, *p* = 1.50E-10	
	Hour 2: *F*_(1,153)_ = 0.026, *p* = 0.871	
	Hour 3: *F*_(1,162)_ = 15.3, *p* = 1.35E-4	
	Hour 4: *F*_(1,165)_ = 12.1, *p* = 6.41E-4	
	Hour 5: *F*_(1,169)_ = 74.4, *p* = 4.52E-15	
	Hour 6: *F*_(1,172)_ = 235.2, *p* = 5.21E-34	
	Hour 7: *F*_(1,164)_ = 358.2, *p* = 4.25E-43	
	Hour 8: *F*_(1,162)_ = 361.4, *p* = 4.18E-43	
	Hour 9: *F*_(1,163)_ = 382.2, *p* = 1.37E-44	
	Hour 10: *F*_(1,172)_ = 135.7, *p* = 1.72E-23	
	Hour 11: *F*_(1,152)_ = 166.7, *p* = 3.26E-26	
	Hour 12: *F*_(1,153)_ = 122.6, *p* = 2.66E-21	
4*E*	One-way ANOVA	Bonferroni's correction for multiple comparisons
	S. basal vs NA22, serotonin, and diacetyl: *F*_(3,417)_ = 584.8	S. basal vs NA22: *p* = 2.83E-123 S. basal vs serotonin: *p* = 4.21E-123 S. basal vs diacetyl: *p* = 3.13E-08
4*F*	One-way ANOVA	Bonferroni's correction for multiple comparisons
	Hour 1: *F*_(3,368)_ = 57.9	S. basal vs NA22: *p* = 2.72E-21 S. basal vs serotonin: *p* = 5.15E-20 S. basal vs diacetyl: *p* = 0.22
	Hour 2: *F*_(3,369)_ = 39.5	S. basal vs NA22: *p* = 1.67E-06 S. basal vs serotonin: *p* = 0.027 S. basal vs diacetyl: *p* = 3.74E-07
	Hour 3: *F*_(3,394)_ = 33.6	S. basal vs NA22: *p* = 1.34E-05 S. basal vs serotonin: *p* = 0.039 S. basal vs diacetyl: *p* = 5.40E-06
	Hour 4: *F*_(3,393)_ = 34.5	S. basal vs NA22: *p* = 9.22E-18 S. basal vs serotonin: *p* = 1.06E-11 S. basal vs diacetyl: *p* = 0.0012
	Hour 5: *F*_(3,396)_ = 57.2	S. basal vs NA22: *p* = 1.37E-24 S. basal vs serotonin: *p* = 1.58E-22 S. basal vs diacetyl: *p* = 2.04E-14
	Hour 6: *F*_(3,397)_ = 66.4	S. basal vs NA22: *p* = 2.54E-25 S. basal vs serotonin: *p* = 8.96E-29 S. basal vs diacetyl: *p* = 4.40E-13
	Hour 7: *F*_(3,397)_ = 104.8	S. basal vs NA22: *p* = 8.01E-36 S. basal vs serotonin: *p* = 6.23E-42 S. basal vs diacetyl: *p* = 6.29E-14
	Hour 8: *F*_(3,395)_ = 132.6	S. basal vs NA22: *p* = 5.78E-42 S. basal vs serotonin: *p* = 4.70E-51 S. basal vs diacetyl: *p* = 8.84E-26
	Hour 9: *F*_(3,398)_ = 141.3	S. basal vs NA22: *p* = 1.15E-48 S. basal vs serotonin: *p* = 6.19E-47 S. basal vs diacetyl: *p* = 1.61E-10
	Hour 10: *F*_(3,386)_ = 109.6	S. basal vs NA22: *p* = 2.24E-42 S. basal vs serotonin: *p* = 6.76E-27 S. basal vs diacetyl: *p* = 0.029
	Hour 11: *F*_(3,387)_ = 165.4	S. basal vs NA22: *p* = 2.20E-47 S. basal vs serotonin: *p* = 3.00E-26 S. basal vs diacetyl: *p* = 0.061
	Hour 12: *F*_(3,387)_ = 191.8	S. basal vs NA22: *p* = 2.50E-39 S. basal vs serotonin: *p* = 6.67E-27 S. basal vs diacetyl: *p* = 1.07E-09
5*B*	One-way ANOVA	Bonferroni's correction for multiple comparisons
	N2 vs *odr-10*, *tax-4*, and *mec-4* : *F*_(3,207)_ = 62.5	N2 vs *odr-10*: *p* = 2.93E-11 N2 vs *tax-4*: *p* = 2.66E-05 N2 vs *mec-4*: *p* = 0.026
5*C*	One-way ANOVA	Bonferroni's correction for multiple comparisons
	Hour 1: *F*_(3,191)_ = 21.0	N2 vs *odr-10*: *p* = 0.089 N2 vs *tax-4*: *p* = 6.93E-06 N2 vs *mec-4*: *p* = 1
	Hour 2: *F*_(3,188)_ = 8.81	N2 vs *odr-10*: *p* = 0.171 N2 vs *tax-4*: *p* = 1 N2 vs *mec-4*: *p* = 0.0076
	Hour 3: *F*_(3,192)_ = 2.87	N2 vs *odr-10*: *p* = 1 N2 vs *tax-4*: *p* = 1 N2 vs *mec-4*: *p* = 0.049
	Hour 4: *F*_(3,197)_ = 6.57	N2 vs *odr-10*: *p* = 1 N2 vs *tax-4*: *p* = 0.039 N2 vs *mec-4*: *p* = 0.0024
	Hour 5: *F*_(3,196)_ = 25.1	N2 vs *odr-10*: *p* = 2.63E-07 N2 vs *tax-4*: *p* = 1 N2 vs *mec-4*: *p* = 0.255
	Hour 6: *F*_(3,199)_ = 34.9	N2 vs *odr-10*: *p* = 2.36E-12 N2 vs *tax-4*: *p* = 1 N2 vs *mec-4*: *p* = 1
	Hour 7: *F*_(3,201)_ = 33.3	N2 vs *odr-10*: *p* = 6.91E-08 N2 vs *tax-4*: *p* = 0.078 N2 vs *mec-4*: *p* = 0.493
	Hour 8: *F*_(3,201)_ = 34.7	N2 vs *odr-10*: *p* = 7.42E-09 N2 vs *tax-4*: *p* = 1 N2 vs *mec-4*: *p* = 0.072
	Hour 9: *F*_(3,197)_ = 21.2	N2 vs *odr-10*: *p* = 1 N2 vs *tax-4*: *p* = 0.0053 N2 vs *mec-4*: *p* = 1.06E-06
	Hour 10: *F*_(3,192)_ = 16.2	N2 vs *odr-10*: *p* = 0.426 N2 vs *tax-4*: *p* = 0.0064 N2 vs *mec-4*: *p* = 8.39E-4
	Hour 11: *F*_(3,194)_ = 8.41	N2 vs *odr-10*: *p* = 0.294 N2 vs *tax-4*: *p* = 0.111 N2 vs *mec-4*: *p* = 0.680
	Hour 12: *F*_(3,194)_ = 3.98	N2 vs *odr-10*: *p* = 0.848 N2 vs *tax-4*: *p* = 0.500 N2 vs *mec-4*: *p* = 1
5*E*	One-way ANOVA	
	*F*_(1,94)_ = 180.0, *p* = 1.47E-23	
5*F*	One-way ANOVA	
	Hour 1: *F*_(1,88)_ = 0.032, *p* = 0.857	
	Hour 2: *F*_(1,86)_ = 7.36, *p* = 0.008	
	Hour 3: *F*_(1,84)_ = 45.4, *p* = 1.91E-09	
	Hour 4: *F*_(1,90)_ = 21.6, *p* = 1.14E-05	
	Hour 5: *F*_(1,90)_ = 11.7, *p* = 9.53E-04	
	Hour 6: *F*_(1,89)_ = 12.4, *p* = 6.93E-04	
	Hour 7: *F*_(1,88)_ = 5.53, *p* = 0.021	
	Hour 8: *F*_(1,92)_ = 10.1, *p* = 0.002	
	Hour 9: *F*_(1,92)_ = 18.8, *p* = 3.69E-05	
	Hour 10: *F*_(1,87)_ = 24.4, *p* = 3.69E-06	
	Hour 11: *F*_(1,88)_ = 18.9, *p* = 3.66E-05	
	Hour 12: *F*_(1,85)_ = 34.5, *p* = 8.00E-08	
7*B*	Unpaired two-tailed *t* test	
	*t* = 4.343; df = 20; *p* = 3.16E-04	
7*C*	Unpaired two-tailed *t* test	
	*t* = −3.694; df = 20; *p* = 0.0014	
8*E*	Linear regression	
	Result: peak df/F_0_ = 3.518 + 0.065*h	
	SEM of slope: 0.049	
	*p* of slope: 0.193	
9*C*	Unpaired two-tailed *t* test	
	*t* = −13.023; df = 28; *p* = 2.11E-13	
9*E*	Unpaired two-tailed *t* test	
	*t* = −5.5; df = 24; *p* = 1.18E-05	
9*F*	Unpaired two-tailed *t* test	
	ASH: *t* = 0.452; df = 33; *p* = 0.654	
	AWA: *t* = −0.146; df = 24; *p* = 0.885	

#### Software accessibility

Software for control systems and data analysis are available on request.

## Results

### High-throughput analysis of adult sleep

**Figure 1.**
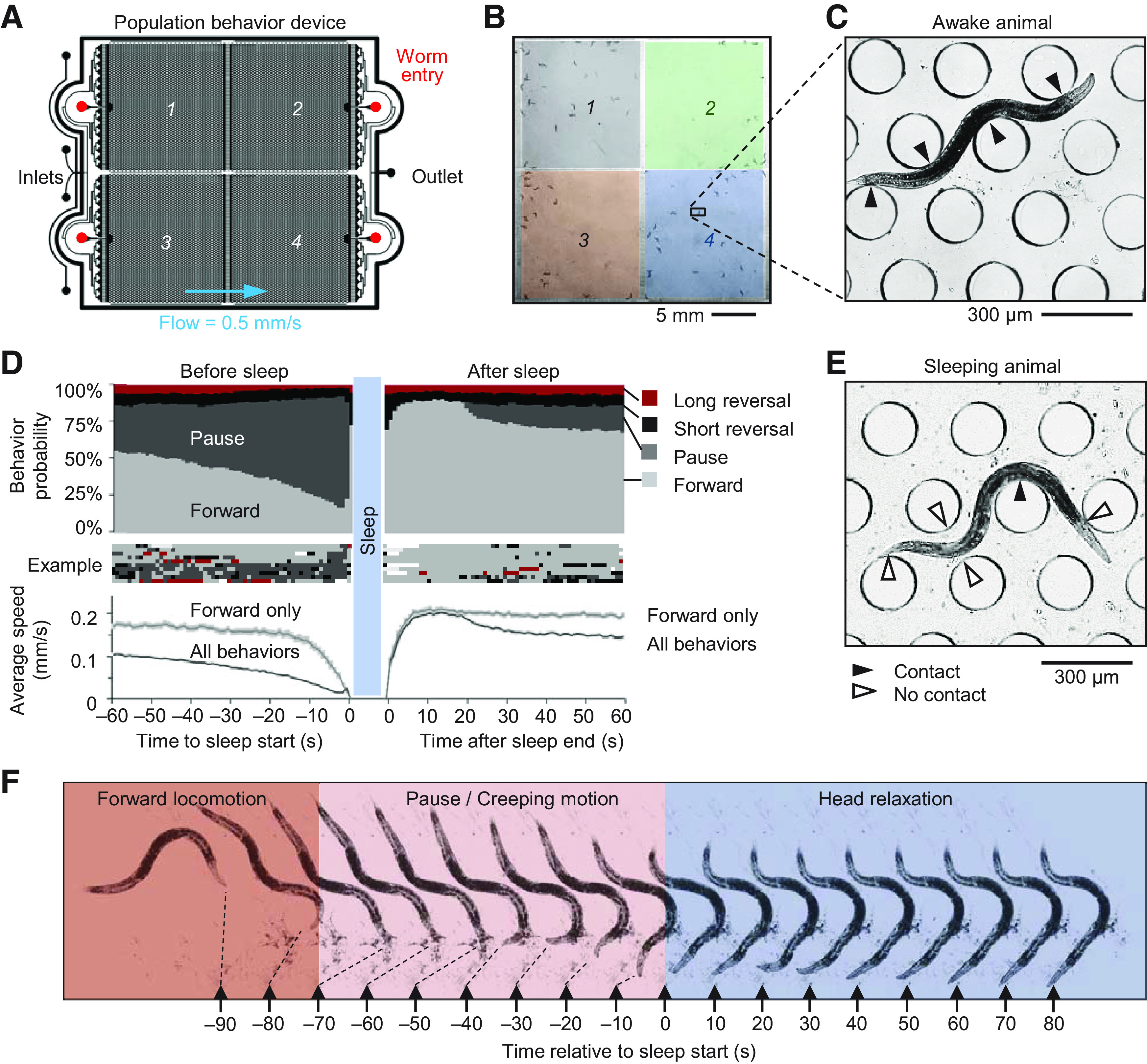
Young adult sleep in wild-type *C. elegans* in “population behavior” microfluidic devices. ***A***, Schematic of the “population behavior” microfluidic device, including multiple inlets to switch fluids, four worm entry ports to introduce separate worm populations, and a flow outlet. ***B***, Image frame of a device containing ∼100 animals, ∼25 in each of four separated 16 × 15 mm arenas. ***C***, Awake animals roam freely between 200-µm diameter microposts. An awake animal exhibits active contact (filled arrows) around posts along the entire body length. ***D***, Distribution of behavior probability and average speed in the 60 s before and after sleep bouts of at least 1 min. Data are from 4359 sleep bouts from 697 wild-type animals over 12 h that had adjacent wake states at least 1 min (32% of total). Error bar shading in average speed plots indicate 95% confidence interval, and “forward only” speed excludes pauses and reverse behaviors. Example of 10 individual events is shown. Accuracy of automated sleep bout prediction is assessed in Table 2. ***E***, A sleeping animal in the microfluidic device exhibits a straight head posture and the relaxed, bent body passively contacts only one to two posts (filled arrow) because of fluid flow, leaving others untouched (open arrows). ***F***, Montage (10-s interval) of an animal transitioning between forward motion (red), pausing/creeping motion (pink) and sleep with characteristic head relaxation (blue). Black triangles represent a fixed position for each image located at the final position of the mouth, and microposts were background subtracted for clarity.

Sleep behavior, defined by periods of behavioral quiescence, was observed in young adult *C. elegans* over 12 h in microfluidic behavior arenas (Albrecht and Bargmann, 2011). Each microfluidic device contained four 16 × 15 mm arenas housing four independent populations of ∼25 animals that share the same dynamic, switchable fluidic environment with continuous flow (Fig. 1*A*,*B*). A hexagonal array of 70-µm-tall microposts enables free sinusoidal crawling behavior as animals gain traction from contact with several microposts along the body (Fig. 1*C*). Wild-type animals roam microfluidic arenas with predominantly forward locomotion, separated by momentary pauses, spontaneous short reversals (<1 s), and long reversals coupled with reorienting omega turns (Gray et al., 2005; Albrecht and Bargmann, 2011). Awake animals may pause briefly to feed if bacterial food is present (Flavell et al., 2013) or when encountering obstacles such as other animals or arena barriers. Other times, animals enter a prolonged quiescence state that lasts between ∼20 s and several minutes (Movie 1). These bouts begin with animals gradually slowing their mean forward locomotion speed over 10–20 s (Fig. 1*D*), often pausing briefly a few times during slow, creeping motion. Animals then gradually adopt a relaxed body posture (Schwarz et al., 2012) over ∼1 min and cease further movement (Fig. 1*E*,*F*). Sleeping animals are apparent visually in microfluidic arenas by their straight head and passive contact with only one to two microposts (Fig. 1*E*), whereas awake animals actively bend around several posts (Fig. 1*C*). After one or more minutes, animals quickly wake and resume forward (or occasionally reverse) locomotion, accelerating to a typical 0.15 mm/s forward velocity within 5 s.

Movie 1. Sleep tracking in “population behavior device.” Video shows three portions of a 12-h video tracking sleep behavior in wild-type animals: early (1.5 h), middle (5.5 h), and late (11 h). Animals detected in a sleep state are circled. Video is accelerated 100×. 10.1523/JNEUROSCI.1701-20.2020.video.1

Since pauses reflect both the extended quiescent states of sleep bouts and the momentary pauses of awake animals, true sleep states were automatically identified by tracking centroid movement filtered by the characteristic duration, history, and body shape of sleep. Using temporal parameters based on sleep entry and exit dynamics (onset after 20-s continuous pausing and ending at 5-s non-pausing), automatic classification of sleep bouts showed 95.2% agreement with human observation, with slight underestimation of sleep states (1.9% false discovery rate; 7.9% false omission rate; *n* = 500 randomly selected bouts; Table 2). These detected sleep bouts excluded the brief pauses that precede a sleep bout, and included momentary “twitch” movements during sleep which can be caused by contact from other animals, flow disturbance, or presumed involuntary movements, and do not signal exit of a sleep state.

**Table 2.** Population behavior device accuracy assessment

	Population behavior device	Observation	
		Sleep	Awake
Prediction	Sleep	255	19
	Awake	5	221
Accuracy	95.2%		
False discovery rate	1.9%		
False omission rate	7.9%		

**Figure 2.**
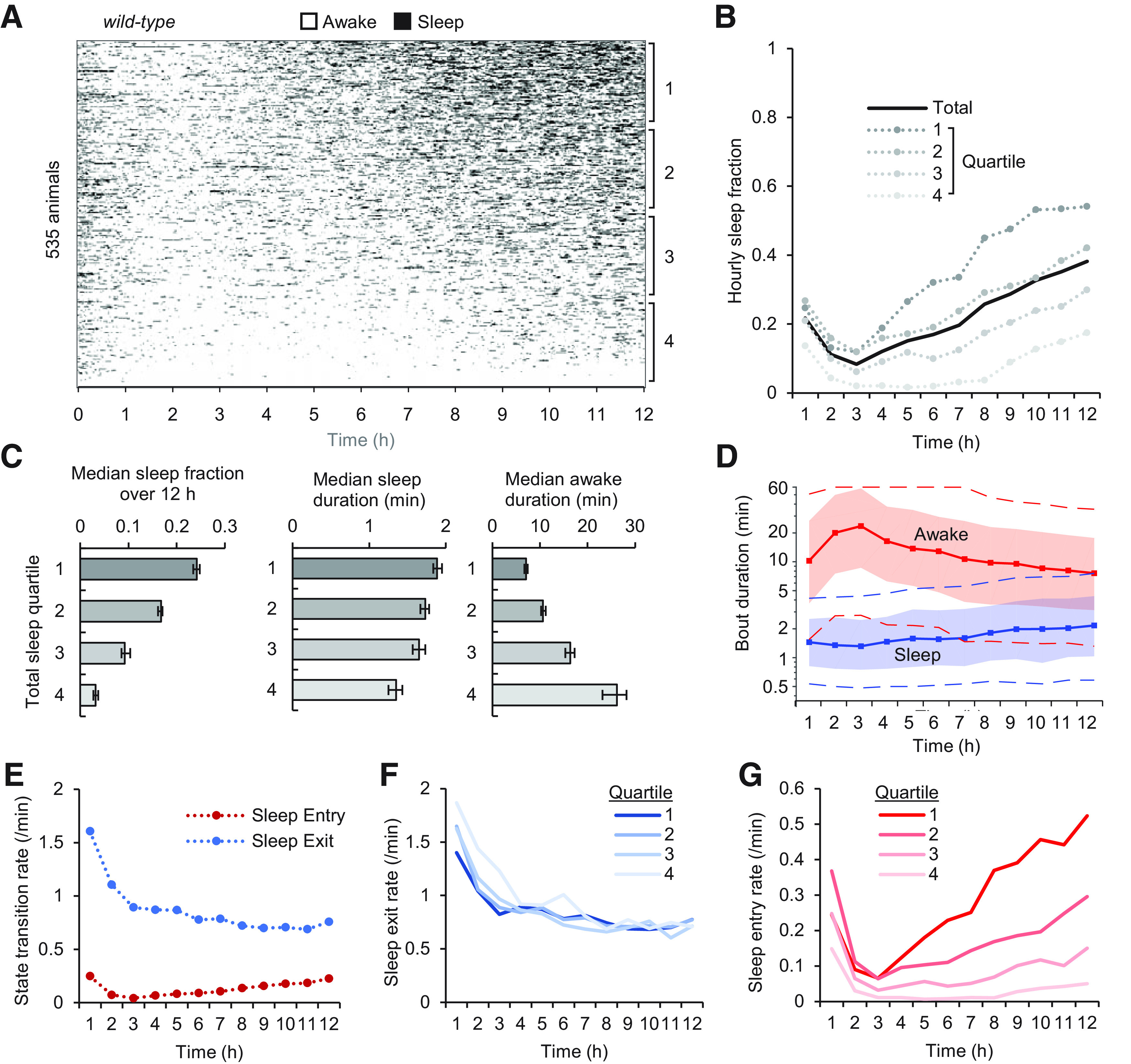
Dynamics of young adult sleep behavior in the microfluidic environment. ***A***, Raster plot of sleep events (black) over 12 h, sorted by total sleep fraction (*n* = 535 animals). ***B***, Hourly sleep fraction for all animals from ***A*** and grouped into four quartiles by their total 12-h sleep fraction (quartile 1 = most sleep). ***C***, Median sleep fraction, sleep bout duration, and awake bout duration from data in ***A***, separated by total sleep quartiles. Error bars indicate 95% confidence interval. ***D***, Changes to sleep behavior over 12 h represented by sleep and awake bout duration. Solid lines represent median durations. Shaded regions represent 25–75% quartile durations and dashed lines represent 10% and 90% decile durations of each respective state. ***E***, Average sleep entry and exit transition rate for each hour of experimentation from data in ***A***. Separating these curves by total sleep quartile demonstrates consistent sleep exit rates (***F***) but large variation in sleep entry rates (***G***) across wild-type animals.

We analyzed 535 wild-type (N2) animals for 12 h in continuous slow (0.5 mm/s) flow of S. basal buffer (Fig. 2*A*). Hourly sleep fraction, defined by the fraction of time the animal spends in a sleep state during each hour, decreased on average across the population from 22 ± 0.8% SEM in the first hour to 8 ± 0.5% in hour 3, then increased steadily to 38 ± 1% in hour 12 (Fig. 2*B*). A wide range of sleep behavior was observed among individual wild-type animals, with 95% exhibiting a 12-h total sleep fraction ranging from 4% to 43%. To assess variability in sleep dynamics, we divided animals into quartiles by total sleep fraction. Sleep dynamics were similar in all quartiles, with sleep fraction increasing over time after 3 h (Fig. 2*B*), but median sleep fraction over 12 h varied greatly across quartiles from 3% to 24%. Median sleep duration remained between 1.3 and 1.9 min for each quartile (Fig. 2*C*), whereas median awake duration varied more greatly, with the top quartile of sleeping animals remaining awake for a median of 7 min, about one-quarter of the most active animals (27 min awake). The increase in sleep fraction from hours 3–12 resulted from longer sleep bouts and shorter awake periods (Fig. 2*D*). These changes were associated with both an increased rate of sleep entry (more sleep pressure) and a decreased rate of sleep exit (more sleepiness; Fig. 2*E*). The rate of sleep exit remained consistent across total sleep quartiles (Fig. 2*F*), while the rate of sleep entry varied greatly (Fig. 2*G*). Together, these results indicate that individual sleep bouts were similar across wild-type animals, whereas variability in sleep fraction across the population predominantly arose because of variation in frequency of sleep bouts, or equivalently, to variation in the rate of sleep entry and in the duration of awake bouts.

### Environmental and sensory effects on sleep dynamics

**Figure 3.**
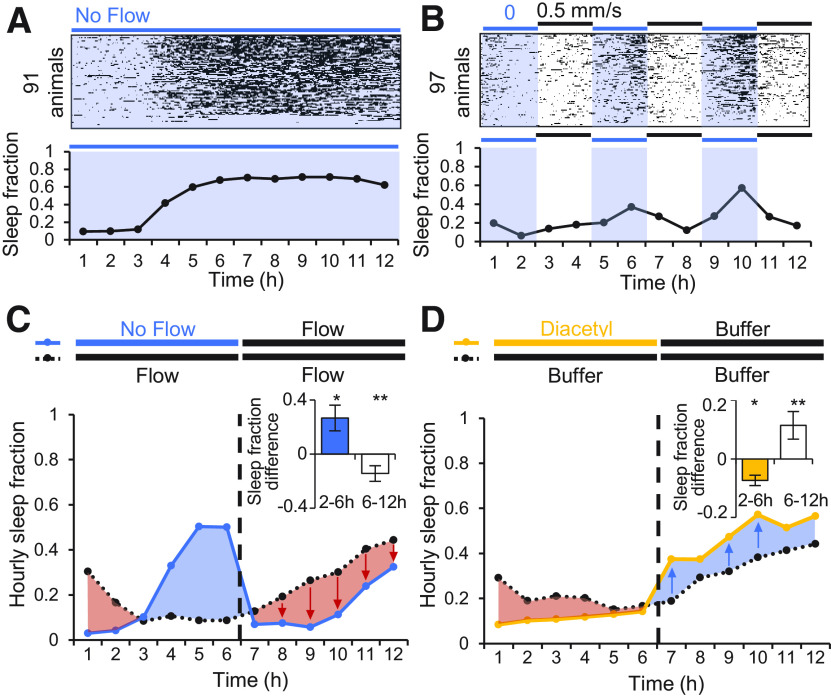
Effects of fluid flow and sleep perturbation. ***A***, Effect of stationary fluid on sleep behavior over 12 h (*n* = 91 animals). Raster plot of sleep events shown above, and mean hourly sleep fraction below. ***B***, Effect of pulsed buffer flow on sleep behavior over 12 h (*n* = 97 animals). Fluid flow alternated between no flow and moderate flow (0.5 mm/s) every 2 h. Raster plot of sleep events shown above, and mean hourly sleep fraction below. ***C***, Increased sleep during 6 h of static flow suppressed sleep in six subsequent hours of buffer flow (blue) compared with continuous flow controls (black). Plot inset compares average hourly difference in sleep fraction between the variable flow group and constant flow control in hours 2–6 (flow effect) and in hours 6–12 (sleep compensation effect). ***D***, Decreased sleep during 6 h of 1.1 μm diacetyl stimulation increased sleep in six subsequent hours of buffer flow (yellow) compared with buffer controls (black). Plot inset compares average hourly difference in sleep fraction as in ***C***, inset. Statistics were performed using one-way ANOVA with Bonferroni's correction for multiple comparisons; *p < 0.05, **p < 0.0001.

Sleep entry and exit are sensitive to environmental conditions and sensory input. To test the role of sensory input on sleep, we first assessed the effect of fluid flow in the microfluidic environment, comparing sleep amounts with a slow flow rate (0.5 mm/s), no flow, and periodic pulsing of flow conditions (Fig. 3*A*,*B*). Without flow, sleep fraction was similar to moderate flow conditions for the first 3 h, but rose dramatically from 12% to 42% around 4 h and remained high (∼70%) for the duration of the 12 h experiment (Fig. 3*A*). To test whether resumption of flow would return sleep fraction to baseline rates, we pulsed flow every 2 h, alternating between 0.5 mm/s flow or no flow. Again, sleep fraction remained low for the first 3 h regardless of flow condition, then increased during each flow stoppage after ∼30 min and decreased sharply when flow resumed (Fig. 3*B*).

Sleep fraction when flow resumed fell below measurements in continuous flow, suggesting evidence of a homeostatic sleep mechanism, in which periods of elevated sleep are followed by reduced sleep, and vice versa. We tested this further by subjecting animals to a no flow condition for 6 h, during which they slept more (+26.8 ± 9.5%, *p* = 0.03, *t* test) than control animals in continuous flow after 2 h of acclimation (Fig. 3*C*). Upon resumption of flow, these animals then slept significantly less (−14.3 ± 5.8%, *p* = 8.13 × 10^−5^, *t* test) than control animals over the following 6 h. Conversely, reducing sleep with 1.1 μm diacetyl (−7.4 ± 1.8%, *p* = 0.031, *t* test) resulted in a compensatory increase (+11.5 ± 4.7%, *p* = 1.84 × 10^−5^, *t* test) in sleep fraction compared with controls that persisted for several hours (Fig. 3*D*). Together, these results demonstrate a bidirectional homeostatic sleep response.

**Figure 4.**
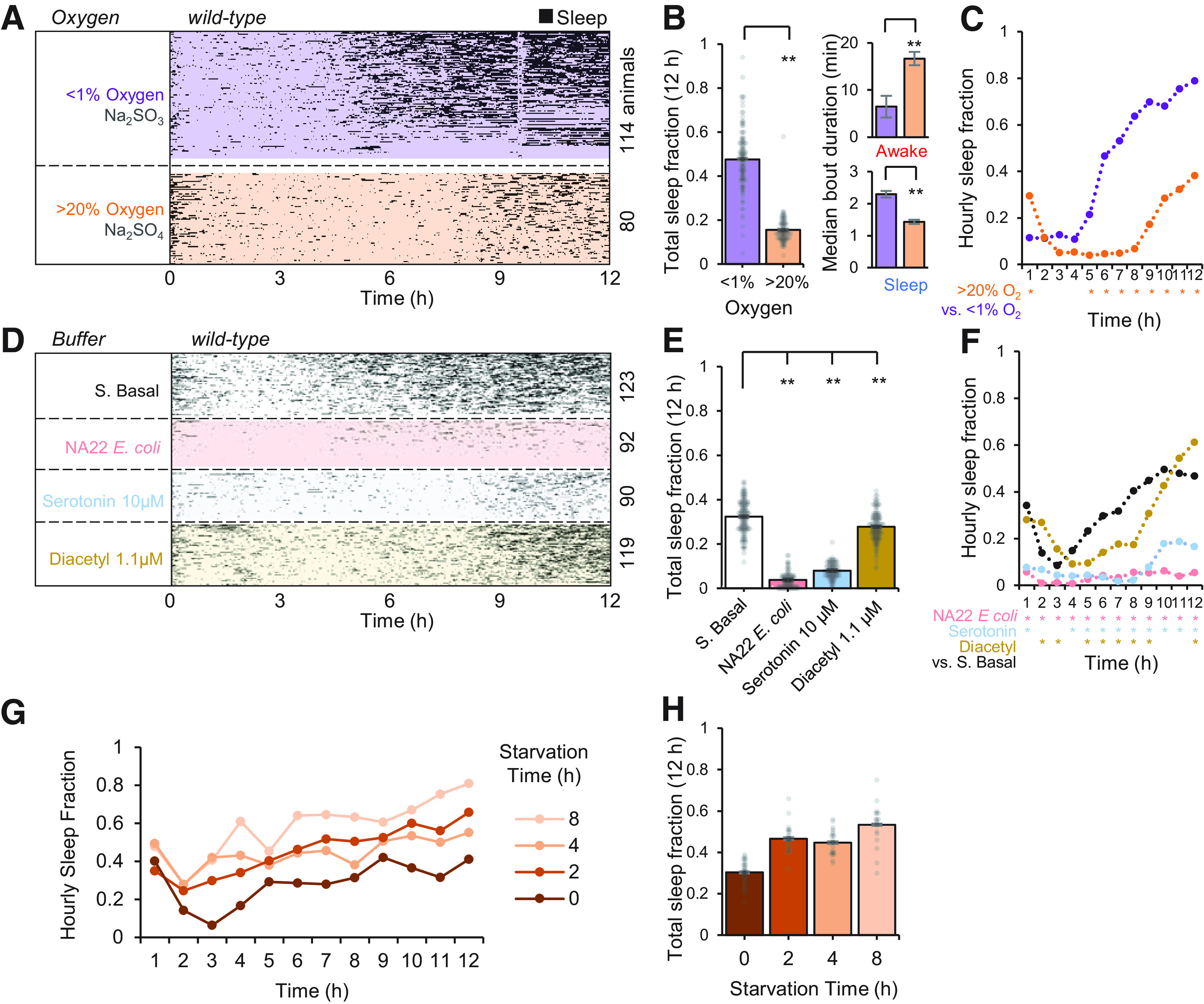
Oxygen and feeding state impact adult sleep in the microfluidic environment. ***A***, Effect of hypoxia on sleep dynamics (*n* = 80–114 animals). Within each group, animals (raster plot rows) are sorted by total sleep fraction. ***B***, Total sleep fraction over 12 h assessing effect of hypoxia on sleep behavior from ***A*** with bars representing population mean ± SEM and points indicating individual animals. Inset shows median sleep and awake bout duration in low and high oxygen. ***C***, Hourly sleep fraction from data in ***A***. ***D***, Effect of feeding and food-related signals comparing sleep behavior in S. basal buffer, in bacterial food (NA22 *E. coli*, OD600 = 0.35), serotonin 10 μm to mimic feeding response, and a food odor diacetyl 1.1 μm (*n* = 90–123 animals). ***E***, Total sleep fraction over 12 h assessing feeding effect on sleep behavior, as in panel ***B***. ***F***, Hourly sleep fraction from data in ***D***. ***G***, Hourly sleep fraction for wild-type animals prestarved for 0–8 h on agar dishes before loading in the microfluidic device. ***H***, Total sleep fraction over assessing effect of starvation on sleep behavior from ***G*** with bars representing population mean ± SEM and points indicating individual animals. Statistics for all plots were performed using one-way ANOVA with Bonferroni's correction for multiple comparisons. For 12-h total sleep fraction plots and median bout duration plots (***B***, ***E***): **p < 0.0001, *p < 0.05. For hourly sleep fraction (***C***, ***F***), significance is noted as *p < 0.0001 for the indicated hour.

Under static conditions, animals can deplete the microfluidic environment of oxygen (Suda et al., 2005; Huang and Lin, 2018), and hypoxia has been shown to induce sleep behavior (Kim and Jin, 2015) especially in starved animals (Skora et al., 2018). We therefore assessed the role of oxygen in adult sleep in microfluidic devices. With continuous flow of 0.5 mm/s, a hypoxic buffer (<1% O_2_, 30 mm sodium sulfite; Jiang et al., 2011) significantly increased total sleep fraction over 12 h (48 ± 1.1%, *p* = 1.74 × 10^−43^, ANOVA) compared with the same solution reoxygenated to >20% O_2_ (16 ± 1.3%; Fig. 4*A–C*). During hypoxia, 13% of sleep bouts were >10 min long compared with only 3.5% of bouts in the reoxygenated buffer, and 1% of hypoxic sleep bouts lasted over 30 min (Fig. 4*B*). Notably, hypoxia increased sleep fraction only after 4 h in the device (Fig. 4*C*), in line with past results suggesting that starvation and hypoxia work together to promote sleep behavior (Skora et al., 2018). The rapid rise in sleep behavior after 4 h mimicked a similar rise in static no-flow conditions (Fig. 3*A*), suggesting that gentle flow replenishes oxygen to suppress sleep behavior.

Because feeding state impacts arousal (Chao et al., 2004; Ezcurra et al., 2016) and starvation may regulate the impact of hypoxia on sleep (Skora et al., 2018), we next assessed the role of feeding and satiety on adult sleep dynamics within microfluidic chambers (Fig. 4*D–F*). The presence of bacterial food (NA22 *E. coli*) suppressed total 12 h average sleep fraction (3.8 ± 0.6%, *p* = 2.83 × 10^−123^, ANOVA) compared with S. basal control (33 ± 0.5%; Fig. 4*E*). Serotonin, which mimics the feeding response (Horvitz et al., 1982), similarly reduced total sleep fraction (8 ± 1.1%, *p* = 4.21 × 10^−103^, ANOVA) compared with control buffer conditions when presented at a moderate concentration of 10 μm. Whereas bacterial food suppressed sleep continuously for 12 h, serotonin suppressed sleep for the first ∼9 h. Similarly, a moderate behaviorally attractive food odor (Chuang and Collins, 1968; 1.1 μm diacetyl) suppressed total sleep fraction compared with control buffer (28 ± 0.9%, *p* = 3.13 × 10^−8^, ANOVA), although to a lesser extent than food or serotonin. Diacetyl suppressed sleep fraction only up to hour 9, consistent with adaptation to the odor over hours (Matsuura et al., 2009; Larsch et al., 2015; Fig. 4*F*). Animals also slept more when starved longer on a plate without food before entry into the microfluidic environment (Fig. 4*G*,*H*). These results suggest that adult sleep behavior in microfluidic devices is driven in part by feeding state and the perception of hunger.

**Figure 5.**
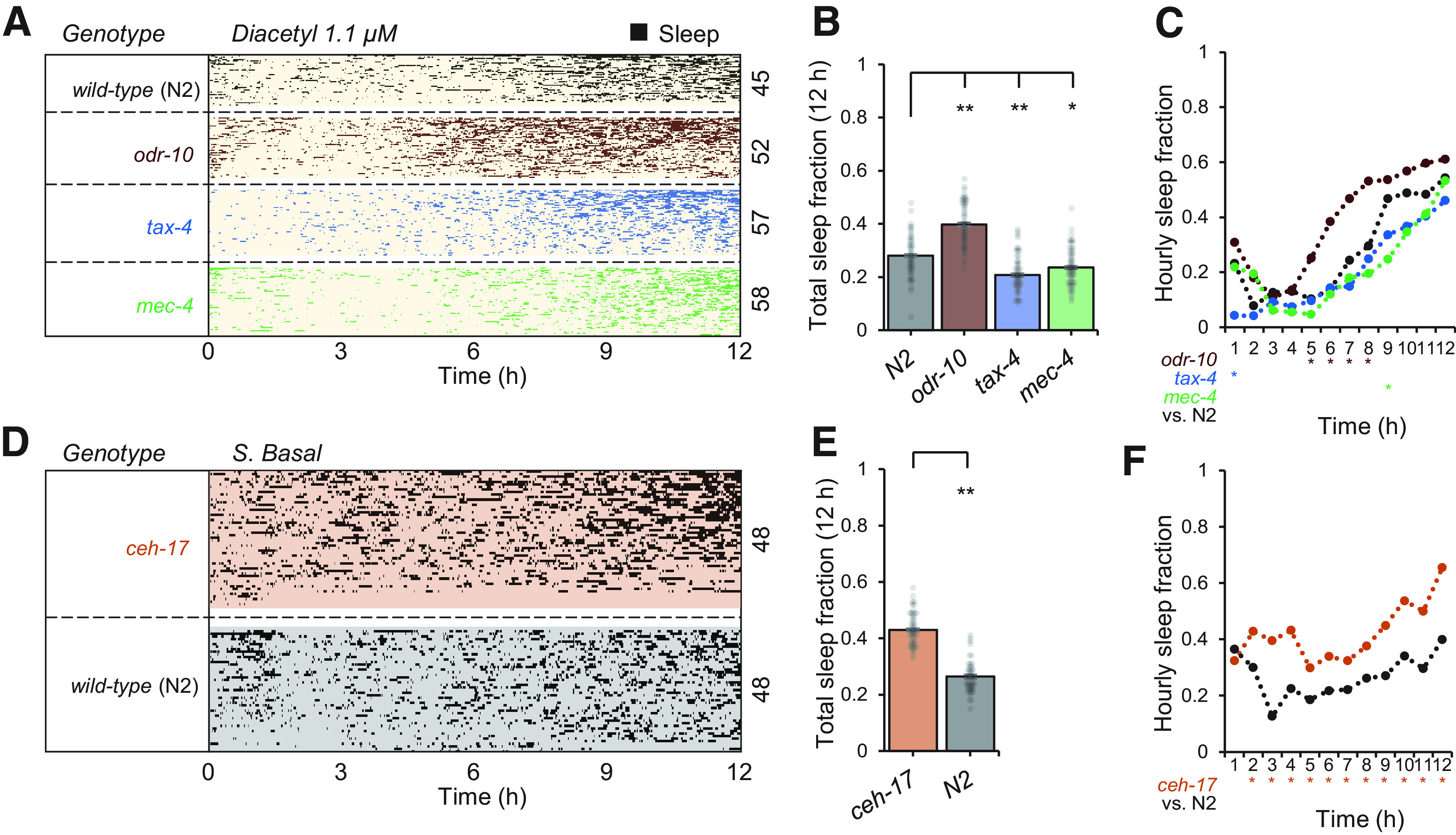
Effect of genetic perturbations on adult sleep. ***A***, Sleep behavior assessed in 1.1 μm diacetyl (*n* = 45–58 animals) in wild-type and sensory mutants affecting diacetyl odor detection (*odr-10*), general sensation (*tax-4*), and light touch (*mec-4*). Within each group, animals (raster plot rows) are sorted by total sleep fraction. ***B***, Total sleep fraction over 12 h assessing effect of sensory mutations on sleep behavior from ***A*** with bars representing population mean ± SEM and points indicating individual animals. ***C***, Hourly sleep fraction from data in ***A***. Extended Data Figure 5-1 compares *odr-10* mutant sleep behavior in diacetyl 1.1 μm with wild-type animals in S. basal buffer. ***D***, *ceh-17* mutants deficient in stress-induced sleep exhibit elevated, not reduced, adult sleep behavior in the microfluidic format (*n* = 48 animals). ***E***, Total sleep fraction over 12 h assessing effect of *ceh-17* mutation on sleep, as in panel ***B***. ***F***, Hourly sleep fraction from data in ***D***. Statistics for all plots were performed using one-way ANOVA with Bonferroni's correction for multiple comparisons. For 12-h total sleep fraction plots (***B***, ***E***): **p < 0.0001, *p < 0.05. For hourly sleep fraction (***C***, ***F***), significance is noted as *p < 0.0001 for the indicated hour.

To observe how sensory information influences sleep, we tested wild-type animals and three sensory mutants (Fig. 5*A–C*) loaded into separate arenas of each “population behavior” device (Fig. 1*A*). Since the odorant diacetyl reduced sleep (Fig. 4*F*), we tested *odr-10* mutants, which lack the diacetyl receptor normally present in the AWA sensory neurons and should not perceive this odor. In the presence of 1.1 μm diacetyl, *odr-10* mutants exhibited a higher total sleep fraction (40 ± 1.0%, *p* = 2.93 × 10^−11^, ANOVA) compared with wild-type (28 ± 1.2%; Fig. 5*B*), and similar to wild-type animals in control buffer conditions lacking the odor (Extended Data Fig. 5-1). In diacetyl, *odr-10* mutants showed a significant increase in hourly sleep fraction compared with wild-type only up to 8 h (Fig. 5*C*), after which habituation to the odor may reduce its influence. Sensory deficient *tax-4* mutants lack a cyclic GMP-gated ion channel necessary for signal transduction in many sensory neurons (Komatsu et al., 1996) and are defective in multiple sensory behaviors, failing to respond to temperature or to water-soluble or volatile chemical cues. However, *tax-4* is not present in AWA neurons; hence, diacetyl-mediated sleep suppression should be preserved in this mutant. Indeed, while *tax-4* showed a moderate decrease in total sleep fraction (20.7 ± 1.0%, *p* = 2.66 × 10^−5^, ANOVA) compared with wild-type over 12 h in 1.1 μm diacetyl, no significant differences in hourly sleep fraction were observed except during the first hour (Fig. 5*C*). Strong suppression of early quiescence bouts in hour 1 in *tax-4* animals (4% vs 21%) suggests that sensory information other than from AWA neurons contributes to elevated quiescence in the first hour. Animals transferred into microfluidic devices experience a novel mechanical environment, including gentle touch of the microposts and continuous fluid flow. While gentle touch deficient *mec-4* mutants showed a slightly lower total sleep fraction than wild-type (24 ± 1.0% vs 28 ± 1.2%, *p* = 0.005, ANOVA), *mec-4* mutants had no significant difference in first hour sleep fraction compared with wild-type (18% vs 21%), suggesting that any sensory information leading to elevated initial quiescence did not come from the *mec-4*-expressing touch receptor neurons ALM, AVM, or PLM. Together, these data demonstrate the role of sensory information in sleep regulation, and the testing of multiple mutants at once in multi-arena microfluidic devices to investigate regulators of sleep dynamics.

Stress-induced sleep is altered in *ceh-17* mutants, in which the ALA neurons fail to develop normally (Pujol et al., 2000). These animals are resistant to *lin-3*/EGF-induced sleep (Buskirk and Sternberg, 2010) and exhibit lower levels of quiescence after exposure to stressors such as heat shock, hyperosmosis, alcohol, cold, and toxins (Hill et al., 2014). However, spontaneous adult sleep in *ceh-17* mutants was significantly higher over 12 h (43 ± 0.9%, *p* = 1.47 × 10^−23^, ANOVA) compared with wild-type (26.5 ± 0.9%) animals in unrestrained microfluidic arenas (Fig. 5*D–F*), suggesting that it differs from ALA-dependent stress-induced sleep.

10.1523/JNEUROSCI.1701-20.2020.f5-1 Extended Data Figure 5-1 Hourly sleep fraction comparison between *odr-10* mutants under constant 1.1 μm diacetyl exposure to wild-type N2 animals in S. basal buffer. *odr-10* mutants lack the diacetyl sensory receptor. N2 data from Figure 2*B*; *odr-10* data from Figure 5*C*. Download Figure 5-1, EPS file.

### Automatic sleep tracking, chemical stimulation, and neural imaging

**Figure 6.**
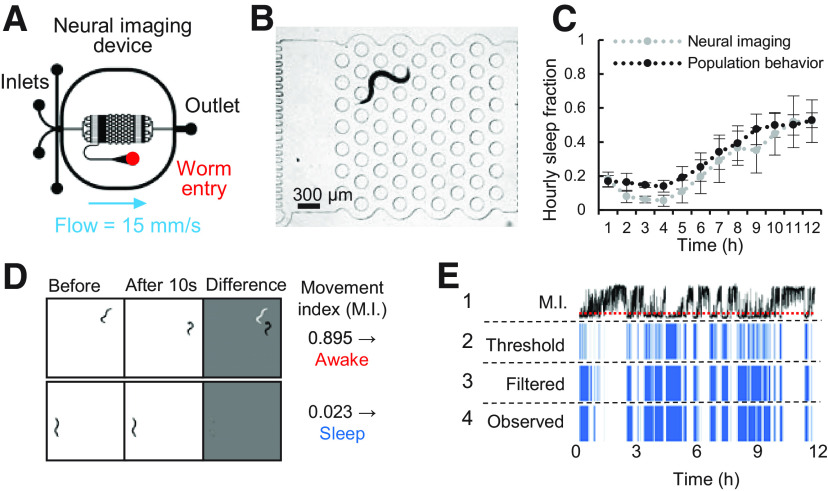
Measuring sleep in “neural imaging” microfluidic devices. ***A***, Design of microfluidic device for closed-loop sleep assessment, chemical stimulation, and neural imaging. Device contains a single 3 × 3 mm arena. ***B***, Sleep is detected in individual animals using pulsed brightfield illumination (λ = 520–550 nm). An awake animal is shown. ***C***, Wild-type adult sleep fraction in “neural imaging” device (*n* = 7 animals) is similar to the larger “population behavior” device (*n* = ∼100 animals). ***D***, Examples of the frame subtraction method for sleep detection showing awake and sleep cases. Movement index (M.I.) represents the fraction of body pixels moved between 10-s frame intervals. ***E***, Schematic of sleep decision processing in a single wild-type animal over 12 h in S. basal: (1) M.I. with red dotted line representing threshold of M.I. 0.125; (2) result of threshold M.I. < 0.125; (3) temporal filtering for five consistent state intervals (40 s total); (4) human ground-truth observation. Accuracy of automated sleep bout prediction over 12 h is assessed in Table 3.

To understand how neural activity changes during sleep cycles, we designed a smaller “neural imaging” microfluidic device containing a single 3 × 3 mm arena with the same micropost array as the “population behavior” device (Fig. 6*A*), but sized to fit the entire field of view at 5× magnification on an epifluorescence microscope (Fig. 6*B*). Wild-type *C. elegans* sleep dynamics in the small “neural imaging” device were equivalent to the larger “population behavior” devices, dropping from 18% to 6% over the first 3 h, then steadily rising to 50% by 12 h, despite a faster flow velocity in the neural imaging microfluidic device (15 vs 0.5 mm/s; Fig. 6*C*). Sleep behavior was tracked using brightfield illumination every 10 s, using a frame subtraction algorithm similar to previous methods (Nagy et al., 2014b; Fig. 6*D*,*E*), and correctly identified sleep bouts with 93.4% agreement with human observers (Table 3).

**Table 3.** Neural imaging device accuracy assessment

	Neural imaging device	Observation	
		Sleep	Awake
Prediction	Sleep	1969	144
	Awake	123	1810
Accuracy	93.4%		
False discovery rate	5.9%		
False omission rate	7.4%		

**Figure 7.**
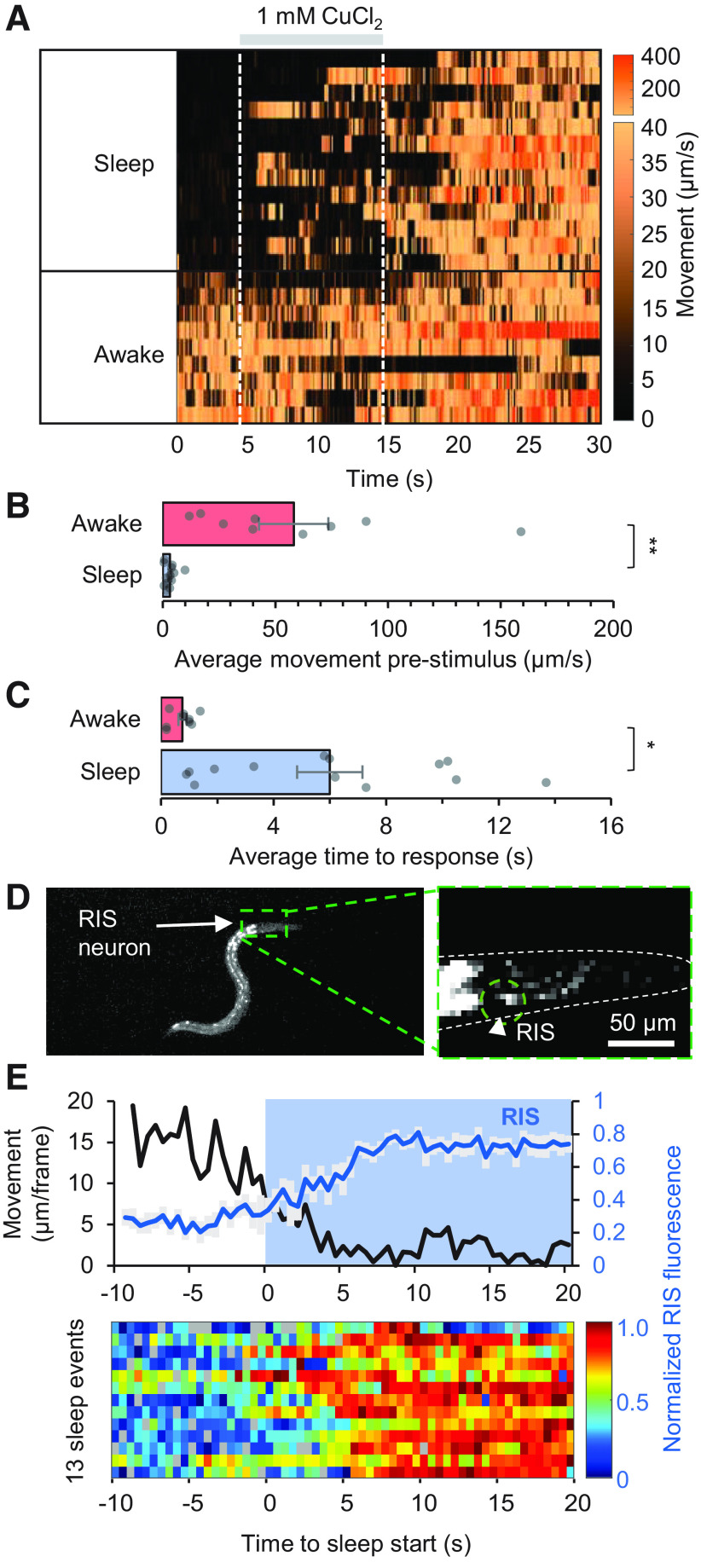
Arousal threshold measurements and sleep-associated neural activity. ***A***, Heatmap showing movement of AIB neuron per frame (0.1-s interval) across 22 pulsed stimulation trials (rows). Animals were stimulated with 1 mm CuCl_2_ from 5 to 15 s during each 30-s trial (gray bar). Data sorted by average movement 5 s before stimulus, indicating the sleep/awake state for each recording. Repeatable microfluidic stimulus onset and removal within <0.5 s is shown in Extended Data Figure 7-1. ***B***, Average movement prestimulus (0–5 s) grouped by sleep state (*n* = 13 sleep, *n* = 9 awake). ***C***, Average time to a reversal or avoidance behavior response for sleeping and awake animals. Statistics for ***B***, ***C*** performed using an unpaired two-tailed *t* test; **p < 0.001, *p < 0.05. ***D***, Image of an animal expressing GCaMP in the RIS neuron. ***E***, Average RIS neuron fluorescence (*n* = 13 sleep events from a single animal) and average neuron centroid movement per frame (0.5-s interval). Neural activity is normalized to minimum and maximum intensity of each RIS neuron trace during the 30 s before and after the awake to sleep transition. Heatmap of all neural recordings is shown below.

The “neural imaging” device provides fast temporal control of chemical stimuli, capable of reproducible fluid switching in <0.5 s (Extended Data Fig. 7-1) without disturbing natural behaviors. We assessed arousal threshold by testing sensory responsiveness of sleeping and awake wild-type animals to aversive 10-s pulses of 1 mm copper chloride solution, recording the time elapsed between chemical onset and the initial reversal movement response. Sleep or wake states were determined by average pixel movement 5 s before stimulation (Fig. 7*A*), which was significantly higher in awake versus sleeping states (58.1 ± 15.3 vs 3.2 ± 0.7 µm/s, *p* = 3.2 × 10^−4^, *t* test; Fig. 7*B*). Reversal responses in a sleep state were about eight times slower (6.0 s ± 1.2 s, *p* = 0.0014, *t* test) than in an awake state (0.76 s ± 0.14 s). This delay is consistent with an increased threshold for sensory responsiveness in sleeping young adult animals (Fig. 7*C*), as has been shown during lethargus to mechanical and chemical stimuli in developmentally-timed sleep (Raizen et al., 2008).

10.1523/JNEUROSCI.1701-20.2020.f7-1 Extended Data Figure 7-1 Microfluidic stimulus onset and removal timing measured by flow of fluorescein dye. Fluorescent pixel intensity switches within <0.5 s during a 10-s dye pulse from 5 to 15 s. Download Figure 7-1, EPS file.

RIS interneuron activity correlates with the onset of developmentally-timed sleep (Maluck et al., 2020; Turek et al., 2013) and quiescent behavior in adults (Steuer Costa et al., 2019). To demonstrate neural imaging during spontaneous sleep-wake cycles in the microfluidic device, we recorded activity in the RIS interneuron expressing GCaMP3 (Fig. 7*D*) in freely moving animals while simultaneously assessing movement behavior. As expected, RIS activity increased at the onset of adult sleep (Fig. 7*E*).

### Closed-loop stimulation and neural imaging of a reversal circuit

**Figure 8.**
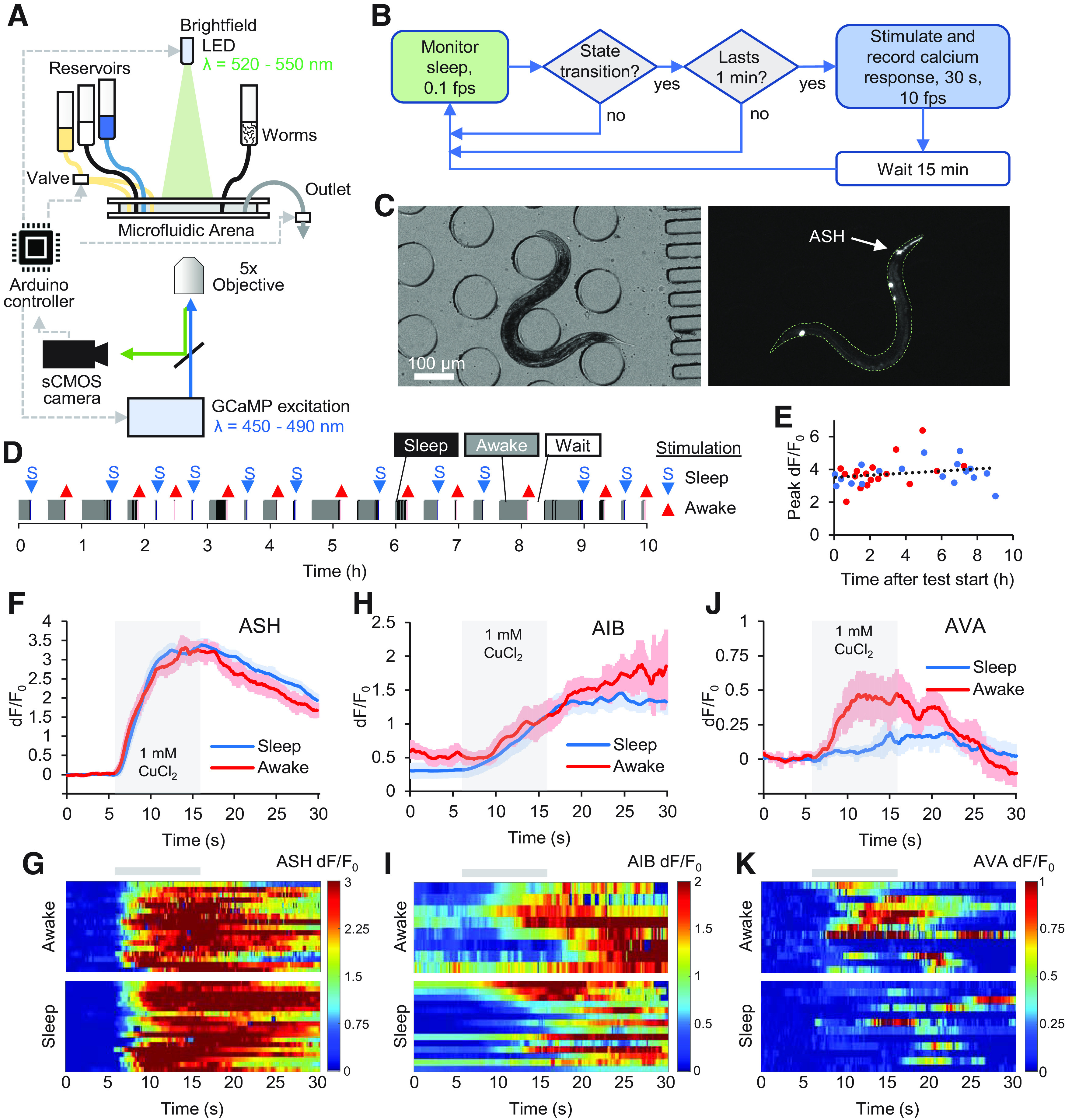
Closed-loop stimulation and neural recording in individual free-behaving animals. ***A***, Schematic of closed-loop neural recording set-up for sleep/awake response tracking. Video recording, valve control, and LED triggering were controlled through an Arduino microcontroller. Brightfield images were used to track sleep behavior and fluorescent images were used measure GCaMP calcium transients. Image capture, sleep/awake determination, and chemical stimulation were controlled by computer in a closed loop without user intervention. ***B***, Decision process schematic of closed-loop experiment. Green (brightfield) and blue (fluorescent) shading of decision nodes indicate corresponding illumination source during frame capture. ***C***, Brightfield (λ = 520–550 nm) and fluorescent (λ = 450–490 nm) images of a freely-moving animal expressing GCaMP in ASH neurons. ***D***, Example showing behavior and neural recording trials in a typical 10-h closed-loop experiment. Behavior patterns and distribution before sleep/wake transitions are shown in Extended Data Figure 8-1. ***E***, Peak ASH neural dF/F_0_ responses to 1 mm CuCl_2_ pulses plotted did not show significant adaptation over 10 h. ***F***, Average ASH neural responses in sleep and awake states to 10-s aversive CuCl_2_ pulses (*n* = 18 sleep, 17 awake). Neural network map downstream of ASH neurons is plotted in Extended Data Figure 8-2. ***G***, Heatmap of individual ASH responses from ***F***. ***H***, Average AIB neural responses in Sleep/Awake states to 10-s CuCl_2_ pulses (*n* = 13 sleep, 8 awake). Extended Data Figure 8-3 shows AIB neural responses aligned to reversal behavior. ***I***, Heatmap of individual AIB responses from ***H***. ***J***, Average AVA neural responses in sleep/awake states to 10-s CuCl_2_ pulses (*n* = 13 sleep, 12 awake). ***K***, Heatmap of individual AVA responses from ***J***.

An increased threshold for sensory responsiveness during sleep suggests sleep-dependent modulation to neural activity in *C. elegans*, either in sensory responses to stimulation, or in downstream interneurons or motor neurons. For example, during lethargus states in developmentally-timed sleep, aversive chemical pulses (1 mm copper chloride) elicited weaker ASH sensory neuron activity (Cho and Sternberg, 2014). However, it is unclear whether sensory-level modulation occurs during adult sleep as well. Since adult sleep is not synchronized across animals, or within an individual, we developed a closed-loop system that monitors sleep state every 10 s and triggers a stimulation and neural recording when user-programmable conditions are met (Fig. 8*A*; Movie 2). Here, we chose to stimulate 1 min after a sleep state transition, allowing a 15-min recovery period between stimulation trials (Fig. 8*B*). Brief pulses of blue light excitation were used for fluorescent imaging to measure calcium activity during each 30-s trial (Fig. 8*C*), as strong blue light can cause arousal by itself (Edwards et al., 2008), and sleep state was monitored by behaviorally-neutral green light.

10.1523/JNEUROSCI.1701-20.2020.f8-1 Extended Data Figure 8-1 Prestimulus behavior history up to 5 min before 22 stimulation captures during closed-loop stimulation of animals entering and exiting sleep bouts, from Figure 8*D*. Average instantaneous sleep fraction is plotted below. Arrowheads represent start of stimulation paradigm in the sleep (blue) or awake (red) state. Download Figure 8-1, EPS file.

10.1523/JNEUROSCI.1701-20.2020.f8-2 Extended Data Figure 8-2 Neural connections linking copper chloride sensation for reversal behavior on arousal by stimulus in sleep and wake states in Figure 8*F–K*. Download Figure 8-2, EPS file.

10.1523/JNEUROSCI.1701-20.2020.f8-3 Extended Data Figure 8-3 AIB interneuron activity aligned to point of first reversal response of individual trials, showing AIB rise precedes the delayed reversal in sleeping animals. Data from Figure 8*H*,*I*. Download Figure 8-3, EPS file.

Movie 2. Example of closed loop system in “neural imaging device.” Video shows an animal expressing GCaMP in the ASH neuron tracked in the closed loop system. First, awake behavior is detected using brightfield illumination and the system applies a stimulus paradigm, 30 s long with a 10-s pulse of 1 µm copper chloride, recording GCaMP fluorescence. Next, a sleep bout is detected and the same stimulus pattern is initiated 1 min after sleep entry. State change detection and stimulus presentation are indicated above. Video is accelerated 37.5× during brightfield behavior and 3× during fluorescent trials. 10.1523/JNEUROSCI.1701-20.2020.video.2

We measured neural responses to 10-s pulses of 1 mm copper chloride in the ASH sensory neurons over 12 h in individual animals. A typical closed-loop experiment with 15-min recovery per stimulation recorded about one sleep and one awake response per hour over >10 h (Fig. 8*D*,*E*; Extended Data Fig. 8-1). ASH neurons responded strongly and consistently to each copper chloride pulse, regardless of sleep or awake state during stimulation (Fig. 8*F*,*G*), and showed no significant sensory adaptation (Fig. 8*E*).

Since ASH chemosensory responses were equivalent in sleep and awake states, the elevated arousal threshold in sleep could result from diminished activity in interneurons, motor neurons, or in the muscles themselves. ASH is directly presynaptic to AVA premotor interneurons, and also has secondary connections through AIB, AVD, and RIC interneurons (Extended Data Fig. 8-2). As AIB shares a gap junction with the sleep-inducing neuron RIS, and ablation of AIB reduces long reversals (Gray et al., 2005), we recorded AIB and AVA neural activity in sleep and awake states in response to 1 mm copper chloride. Neural responses in AIB were not significantly different between awake and sleep states (Fig. 8*H*,*I*). In contrast, animals in sleep states had diminished AVA responses, increasing average relative GCaMP fluorescence 48% when awake and 19% when asleep (*p* = 0.031, *t* test). AVA neural responses were also delayed relative to the copper pulse (Fig. 8*J*,*K*), consistent with delayed and shortened reversal behaviors (Fig. 7*C*). AIB activity often increased before reversal behavior in sleeping animals but coincided with reversal responses in awake animals (Extended Data Fig. 8-3). This suggests a sleep-dependent behavioral delay downstream of (or bypassing) AIB and presynaptic to AVA, that contributes to the apparent arousal threshold increase in sleeping animals.

### Appetitive sensory modulation during sleep

**Figure 9.**
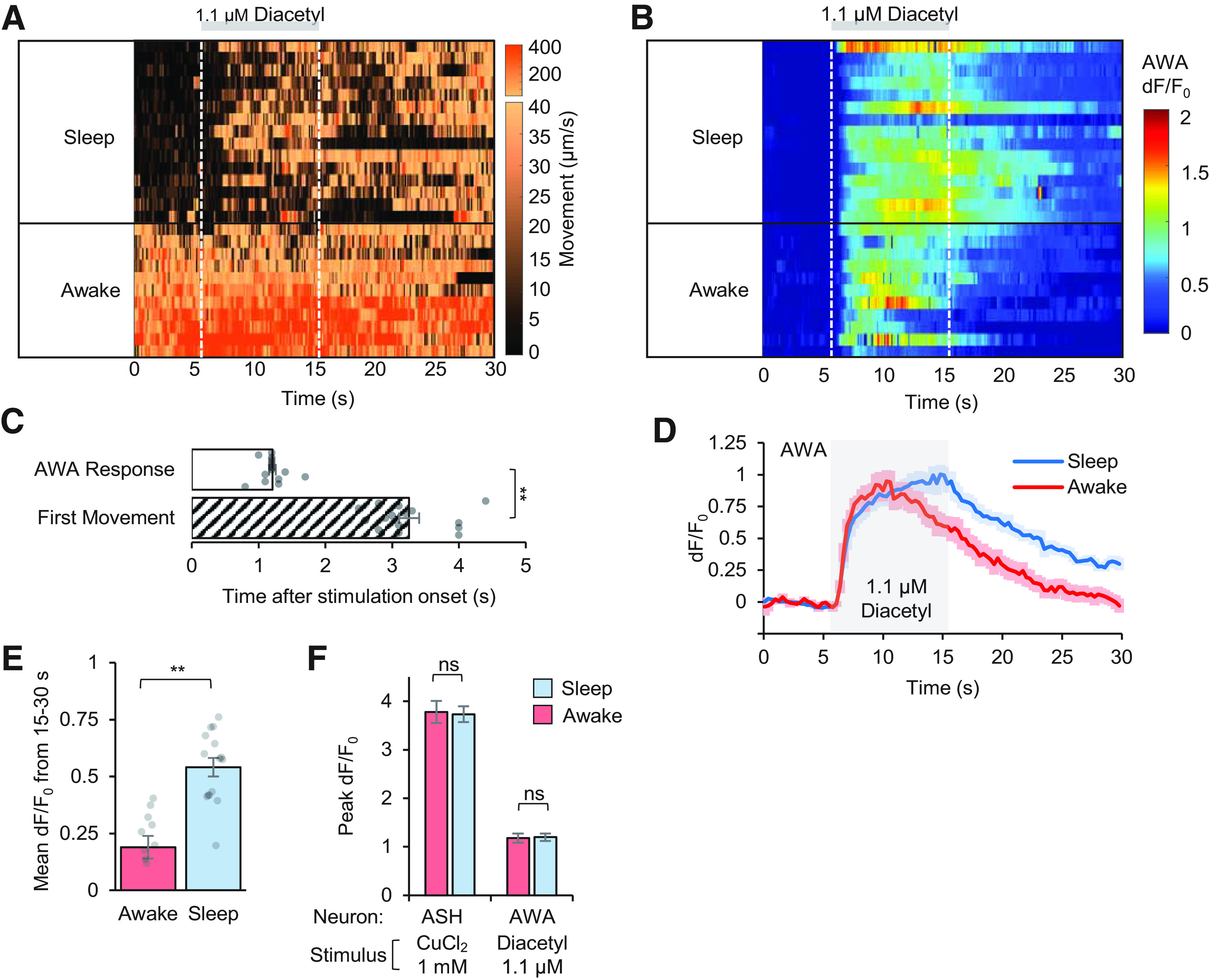
Appetitive sensory neurons show prolonged neural response during sleep. ***A***, Heatmap showing movement of AWA neuron per frame (0.1-s interval) across 26 pulsed stimulation trials (rows) from two animals in separate experiments. Animals were stimulated with 1.1 μm diacetyl between 5 and 15 s during each 30-s trial (gray bar). Data are sorted by average movement 5 s before stimulus, indicating the sleep/awake state for each recording. ***B***, Heatmap of individual dF/F_0_ AWA responses from ***A***. ***C***, Time to first movement in sleeping animals (quantified by time of head movement after stimulus onset), compared with initiation of AWA neural activity (*n* = 15 trials). ***D***, Average dF/F_0_ AWA neural responses in sleep/awake states to 10-s diacetyl pulses from panel ***B*** (*n* = 15 sleep, 11 awake). ***E***, Average dF/F_0_ during 15 s after stimulation indicates prolonged neural response in sleep (*n* = 15 trials) versus awake (*n* = 11 trials) states. ***F***, Peak dF/F_0_ in AWA and ASH neurons in response to stimuli during sleep and awake states (*n* = 26–35 trials per condition). Statistics for ***C***, ***E***, ***F*** performed using an unpaired two-tailed *t* test; **p < 0.001; ns (not significant) *p* > 0.05.

Sleep may affect sensory modalities differently. To compare with ASH aversive response circuits, we assessed sleep-dependent changes in an appetitive sensory circuit using the AWA chemosensory neurons. Whereas aversive stimulation of ASH with copper chloride provoked reversal behavior in both sleeping and awake animals, appetitive stimulation of AWA with 1.1 μm diacetyl elicited slight head movement in sleeping animals and promoted a continuation of forward locomotion behavior in awake animals (Fig. 9*A*). Since awake animals experienced no strong behavior change on presentation of appetitive stimuli, sleep-dependent arousal timing differences could not be made. However, simultaneous measurements of neural activity revealed sleep-dependent differences in neural response (Fig. 9*B*).

Animals that were sleeping before diacetyl stimulation responded with a ∼2 s delay in initial movement (3.25 ± 0.15 s after stimulation onset, *p* = 2.11 × 10^−13^, *t* test) compared with the initial rise in AWA activity (1.21 ± 0.05 s after stimulation onset; Fig. 9*C*). This sleep-dependent response delay was shorter and more consistent than with copper chloride stimulation. AWA neural activity arose equally in awake and sleeping animals at stimulus onset (Fig. 9*B*,*D*), but AWA activity remained high throughout the 10-s diacetyl pulse in sleeping animals, whereas it declined ∼5 s earlier in awake animals (Fig. 9*D*). As a result, AWA neural responses were significantly elevated during the 15 s after stimulation in sleeping animals (0.54 ± 0.04 dF/F_0_, *p* = 1.18 × 10^−5^, *t* test) than awake animals (0.19 ± 0.05 dF/F_0_; Fig. 9*E*). While sleep prolonged sensory neural dynamics in AWA to 1.1 μm diacetyl, peak response levels were unchanged across sleep and awake states, as were ASH responses to 1 mm copper chloride (Fig. 9*F*).

## Discussion

*C. elegans* sleep has been studied previously during developmentally-timed transitions (lethargus) and after induction by satiety or various stresses, but spontaneous adult sleep has been technically more difficult to assess. Adult quiescence behavior in our microfluidic arena devices displays the same general characteristics of sleep previously used to define quiescent behavior as sleep in *C. elegans* during developmentally-timed sleep and stress-induced sleep. Quiescent adults exhibited: (1) an increase in arousal threshold to an aversive chemical stimulus by a delay in behavioral response (Fig. 7*C*); (2) rapid sleep reversibility on changes in fluid flow (Fig. 3*B*); (3) a characteristic relaxed posture (Fig. 1*E*,*F*); and (4) a homeostatic sleep response (Fig. 3*C*,*D*). We observed some differences in unrestrained adult sleep behavior compared with recent reports on adult sleep in open and constrained environments, which we attribute to microfluidic geometry and experiment duration. For example, static fluids and hypoxia were highly somnolent in freely-behaving animals over 12 h, whereas constricted animals increased sleep during gentle microfluidic flow, with no effect of oxygen over 1 h (Gonzales et al., 2019). Spontaneous adult sleep was elevated in *ceh-17* mutant animals, which are deficient in stress-induced sleep, suggesting that spontaneous adult sleep in unrestrictive microfluidic devices is unique to the sleep states previously observed.

Sleep and hunger are mutually inhibitory. In mammals, the hunger-associated peptide ghrelin suppresses sleep, whereas satiety-related leptin and insulin promote sleep (Goldstein et al., 2018). In *C. elegans*, adult sleep behavior was also strongly suppressed by continuous food presentation for the entire 12-h experiment duration. In contrast, well-fed animals introduced into buffer without food gradually increased their sleep fraction over several hours, and prestarvation commensurately accelerated this timing. Presenting exogenous serotonin to mimic the feeding response, or the food odor diacetyl, suppressed sleep for 8–9 h, consistent with adaptation timing to these food-related signals.

Sleep behavior is also sensitive to environmental conditions presented in microfluidic devices. For example, fluid flow in the microfluidic environment is important for maintaining a fresh and constant environment, and cessation of flow increased sleep behavior dramatically after several hours. Static fluid conditions decrease mechanical stimulation, deplete nutrients and oxygen, and increase concentrations of byproducts and CO_2_. Oxygen depletion by animals may be a primary factor driving elevated sleep in static microfluidic conditions, as sleep dynamics were similar in static fluid and with hypoxic buffer flow. Hypoxia increased sleep behavior only after 4 h in freely-behaving animals, likely because of increasing starvation over this time. Similarly, hypoxia was shown to suppress most spontaneous neural activity across the whole brain of trapped *C. elegans,* but only in starved animals (Skora et al., 2018). In mammals, intermittent hypoxia can cause excessive sleepiness (Sanfilippo-Cohn et al., 2006), but can also cause disturbed and superficial sleep with frequent waking via chemoreceptor reflex pathways (Laszy and Sarkadi, 1990). Thus, there is an interplay between arousing and somnolent environmental cues, in addition to feeding state. Further studies in *C. elegans* may be useful to distinguish between these contrasting hypoxic effects and to understand the role of sleep in regulating metabolic systems.

Sensory neural activity directly modulates sleep. For example, sleep suppression by diacetyl was absent in *odr-10* mutants that lack only the diacetyl receptor and are unable to detect this odor. Sensory information and fluid flow also contribute to the initial elevated sleep behavior seen in the first hour of testing as animals acclimate to the microfluidic environment. The general sensory mutant *tax-4* suppressed first-hour sleep, whereas mechanosensory-deficient *mec-4* animals did not, suggesting that gentle touch of microfluidic structures do not contribute to early sleep behavior. Instead, other *tax-4*-dependent sensation, such as from various thermosensory and chemosensory neurons (Komatsu et al., 1996), may be involved in detecting the novel microfluidic environment.

We compared activity of several neurons during sleep and awake states of unrestrained animals. The RIS interneuron is active at the onset of spontaneous adult sleep, as has been shown during developmentally-timed lethargus sleep (Turek et al., 2013; Maluck et al., 2020). The automated closed-loop stimulation system, which requires no user input, further allows unbiased comparison of stimulus-evoked neural responses during alternating sleep and awake bouts within the same animal. The ability to record state-dependent response differences within individuals is particularly important because of the wide variation in sleep dynamics observed across individual animals. Isogenetic animals, even when raised on the same plate from the same parent, exhibited total sleep fractions varying from zero to nearly one half over 12 h. Given the sensitivity of adult sleep to oxygen, feeding state, chemicals, and likely other sensory stimuli, it is possible that animals cultured identically experience slight variations in these inputs. Longitudinal studies capturing dozens of events per animal allow identification of intra-animal differences in sensory processing regardless of population-wide variation in sleep patterns.

An increased arousal threshold in sleeping animals suggests modulation to sensorimotor neural circuit activity in *C. elegans* during sleep. Responses of the AVA command interneurons, which are required for backward locomotion (Zheng et al., 1999; Gray et al., 2005; Piggott et al., 2011) were indeed diminished and delayed during adult sleep, coinciding with delayed behavioral responses. Similarly, diminished AVA activity was previously observed during lethargus (Cho and Sternberg, 2014). However, sensory responses in ASH neurons were not modulated by sleep state in adults, in contrast to the weaker ASH responses observed in larval stages during developmentally-timed sleep (Cho and Sternberg, 2014), suggesting that spontaneous adult sleep is a distinct phenomenon. The first layer AIB interneurons, which share synaptic connections with ASH, the AVA command interneurons, and the RIS sleep-induction neuron, also showed no sleep-dependent difference in response. Together, these results suggest that modulation in sensory processing that leads to reduced arousal response in sleep occurs at or upstream of AVA, such as synaptic signaling from ASH, AIB, or other interneurons (Extended Data Fig. 8-2), or neuropeptides from other sources. One possibility is that sleep increases arousal threshold predominately by diminishing the efficacy of monosynaptic shortcuts to the command interneurons (here, ASH to AVA), whereas sensory information is preserved to first layer interneurons (such as AIB) to allow for rapid arousal from more salient polymodal stimuli from multiple sensory neurons. However, animal survival should benefit from maintaining rapid arousal to potentially harmful stimuli such as sensed by ASH, yet this does not appear true. Alternatively, the dampened brain state apparent in sleep (Nichols et al., 2017) may broadly suppress activity in premotor interneurons like AVA, increasing arousal thresholds equally to all types of sensory input which result in reversal behavior.

But sleep influences sensory modalities differently. The appetitive stimulus diacetyl aroused sleeping animals after several seconds, just as the aversive stimulus, although the stimulus-to-behavior delay was shorter and more consistent (2.5–4.4 s to appetitive forward response vs 1–14 s to aversive reversal response). Further, AWA sensory responses to diacetyl were prolonged in the sleep state, unlike ASH aversive responses. While locomotory feedback is processed by sensorimotor circuits (Hendricks et al., 2012), past studies have shown no AWA response differences between crawling and paralyzed animals (Larsch et al., 2013). This suggests that differences seen in neural response in AWA are because of sleep related mechanisms rather than feedback from locomotion alone. Together, these differences suggest that sleep-dependent circuit modulation acts differently across sensory circuits, and further study of additional sensory stimuli and neurons will be necessary to uncover its architecture and mechanisms.

These flexible microfluidic systems for studying adult sleep in *C. elegans* are applicable to any neuron, stimulus, environment, and genetic perturbation for thorough assessment of sleep behavior and underlying neural responses. For example, it will be informative to compare neural responses in various sleep modes, including hypoxia and starvation-induced sleep as shown here, as well as heat shock and satiety-related sleep. Microfluidic devices are easily customized to different animal sizes by adjusting arena post geometry, for example, to observe L4 animals in lethargus transition stages in developmentally-timed sleep. Other types of oxidative or metabolic stress (such as by chemical oxidants or varying food quality), or sleep disruption via mechanical stimulation or light, can be applied using the same microfluidic devices and tracking methods. Overall, this platform can be used to uncover molecular and neural circuit pathways underlying altered sensation during sleep, toward establishing connections between nematode sleep and associated regulatory mechanisms and human sleep disorders.
